# Should I stay or should I go? Exploring the job preferences of allied health professionals working with people with disability in rural Australia

**DOI:** 10.1186/s12960-015-0047-x

**Published:** 2015-06-30

**Authors:** Gisselle Gallego, Angela Dew, Michelle Lincoln, Anita Bundy, Rebecca Jean Chedid, Kim Bulkeley, Jennie Brentnall, Craig Veitch

**Affiliations:** Centre for Health Research, School of Medicine, University of Western Sydney, Building 3, Campbelltown Campus, Locked Bag 1797, Penrith, New South Wales 2751 Australia; Faculty of Health Sciences, University of Sydney, Cumberland Campus, PO Box 175, East St, Lidcombe, New South Wales 1825 Australia; School of Social Sciences, Faculty of Arts and Social Sciences, UNSW, Sydney, NSW 2052 Australia

**Keywords:** Preferences, Retention, Rural, Disability, Best–worst scaling, Australia, Preferencias, Retención, Rural, Discapacida, Best–worst scaling, Australia

## Abstract

**Introduction:**

The uneven distribution of allied health professionals (AHPs) in rural and remote Australia and other countries is well documented. In Australia, like elsewhere, service delivery to rural and remote communities is complicated because relatively small numbers of clients are dispersed over large geographic areas. This uneven distribution of AHPs impacts significantly on the provision of services particularly in areas of special need such as mental health, aged care and disability services.

**Objective:**

This study aimed to determine the relative importance that AHPs (physiotherapists, occupational therapists, speech pathologists and psychologists – “therapists”) living in a rural area of Australia and working with people with disability, place on different job characteristics and how these may affect their retention.

**Methods:**

A cross-sectional survey was conducted using an online questionnaire distributed to AHPs working with people with disability in a rural area of Australia over a 3-month period. Information was sought about various aspects of the AHPs’ current job, and their workforce preferences were explored using a best–worst scaling discrete choice experiment (BWSDCE). Conditional logistic and latent class regression models were used to determine AHPs’ relative preferences for six different job attributes.

**Results:**

One hundred ninety-nine AHPs completed the survey; response rate was 51 %. Of those, 165 completed the BWSDCE task. For this group of AHPs, “high autonomy of practice” is the most valued attribute level, followed by “travel BWSDCE arrangements: one or less nights away per month”, “travel arrangements: two or three nights away per month” and “adequate access to professional development”. On the other hand, the least valued attribute levels were “travel arrangements: four or more nights per month”, “limited autonomy of practice” and “minimal access to professional development”. Except for “some job flexibility”, all other attributes had a statistical influence on AHPs’ job preference. Preferences differed according to age, marital status and having dependent children.

**Conclusions:**

This study allowed the identification of factors that contribute to AHPs’ employment decisions about staying and working in a rural area. This information can improve job designs in rural areas to increase retention.

## Resumen

**Introducción:**

La distribución desigual de los profesionales de la salud en zonas rurales y distantes está bien documentada. Esto es aún más frecuente en áreas tales como salud mental y servicios para discapacitados. En Australia, al igual que en otros países, la prestación de servicios a las comunidades rurales y distantes se complica aún más debido a que un número relativamente pequeño de personas está dispersos en grandes áreas geográficas.

**Objetivo:**

determinar qué condiciones específicas de empleo influyen en la preferencia declarada de profesionales de la salud (fisioterapeutas, terapeutas ocupacionales, logopedas y psicólogos - “terapeutas”) que viven en una zona rural de Australia y que trabajan con personas con discapacidad.

**Método:**

estudio transversal se llevó a cabo mediante un cuestionario en línea distribuido a terapeutas que trabajan con personas con discapacidad en una zona rural de Australia durante un período de tres meses. Se solicitó información sobre varios aspectos de trabajo actual y sus preferencias se determinaron mediante un experimento de elección discreta de escalas mejor/peor (BWSDCE). Se utilizaron modelos de regresión logística y de clase latente para determinar la importancia relativa de seis atributos de trabajo.

**Resultados:**

Ciento noventa y nueve terapeutas completaron la encuesta; tasa de respuesta fue del 51 %. De ellos 165 completaron el BWSDCE. Para este grupo de profesionales de la salud “autonomía profesional: alta” es el nivel del atributo más valorado, seguido por “planes de viaje: una o menos noches al mes”, “planes de viaje: dos o tres noches por mes” y “acceso adecuado a desarrollo profesional”. Por otro lado los niveles de atributos menos valorados fueron “los arreglos de viaje: cuatro o más noches al mes”, “autonomía profesional: limitada” y “acceso mínimo a desarrollo profesional”. A excepción de “cierta flexibilidad en el trabajo” todos los demás atributos tuvieron una influencia estadística sobre las preferencias de estos profesionales. Las preferencias difieren de acuerdo a la edad, el estado civil y tener hijos a cargo.

**Conclusiones:**

Este estudio permitió la identificación de los factores que contribuyen a la retención de terapeutas en una zona rural. Esta información puede mejorar las políticas de empleo en zonas rurales y distantes para aumentar la retención de estos profesionales en estas zonas.

## Background

A report from the World Health Organization described that access to “well prepared health professionals in sufficient numbers at the right time and right place” is vital to improving health outcomes in rural areas [1]. However, the uneven distribution of allied health professionals (AHPs) in rural and remote Australia and other countries is well documented [2–5]. This is more significant in areas of special need such as mental health, aged care and disability services. In Australia, like elsewhere, service delivery to rural and remote communities is further complicated because relatively small numbers of clients are dispersed over large geographic areas [6].

In Australia, there is no agreed definition of AHPs, and this contributes to the extensive variation in the numbers cited as working within the allied health workforce [7]. In this study, AHP refers to four professional groups: physiotherapists, occupational therapists, speech pathologists and psychologists (AHPs will be used hereafter to refer to this group of professionals). These professions are the main AHPs working in the disability sector and are commonly called “therapists”; they are the focus of this particular study. In Australia the disability sector provides support for people with a broad range of impairments including acquired disabilities such as brain injury and spinal cord injury, irreversible physical injuries and children and adults with intellectual and developmental disabilities (from birth) such as cerebral palsy, autism spectrum disorders and Down syndrome [8].

AHPs assist people with disability to participate fully in family and community life and employment. Delays in access, and poor coordination of services, mean that problems often compound and secondary complications arise, resulting in increased need for services [6]. Unmet needs may also result in reduced participation in family and community life, with flow-on social and economic costs from missed opportunities and lost income [9]. In short, the dearth of rural therapy services in Australia presents a substantial problem.

Greater demand for these AHPs in Australia will be created by the introduction of the National Disability Insurance Scheme (NDIS) which will increase the demand for disability services in the absence of staff to provide these [9]. At the same time, demand will increase due to the ageing of the population, increased life expectancy and population growth. It is predicted that there will be increasing competition across health, disability and community service sectors for scarce human resources [10]. Access to a skilled allied health workforce is vital to maximize the impact of the NDIS at individual, community and national levels [10]. Key Australian rural bodies, service providers, researchers and policymakers are concerned that a demand-driven system will further disadvantage people with disability living in rural areas [10–12].

Dew et al. found that individuals with disability who live in rural and remote areas experience barriers to using individual funding to access therapy via schemes that operate similarly to those proposed under the NDIS. A key barrier is the lack of providers from whom to purchase services [13]. This highlights the importance of attracting and retaining the necessary disability workforce in rural areas. Research to inform workforce policy that supports rural disability service delivery is important given the national shortage of allied health therapy services outside of metropolitan centres [14].

What is known in relation to AHP workforce size and distribution largely takes the form of descriptive statistical data (for example, workforce numbers, characteristics, participation, distribution, trends in recruitment). Recent reports by Health Workforce Australia (HWA) on the physiotherapy and speech pathology workforce conclude that there is no overall shortage in the numbers of AHPs but there is uneven distribution of the workforce with the majority of AHPs living and working in metropolitan or regional centres [15, 16]. HWA notes with regard to the physiotherapy workforce that “push and pull factors and service delivery models for rural and remote areas are areas for investigation for this workforce” (P.42). It is important to note that these reports focus on “mainstream” AHPs and largely ignores “specialist” disability AHPs.

In general, research has shown that the factors responsible for AHP shortages in rural and remote areas include lack of employment options, professional support, limited career structure, social isolation, poor promotion possibilities, ageing of the workforce, low job satisfaction, long hours and travel time [17, 18]. As noted by the Australian Health Workforce Advisory Committee (AHWAC) report [19], the evidence currently used in allied health workforce policy and planning is mainly descriptive. The AHWAC report recommendations include improving data collection and investigating the reasons for “leakage” and low retention of AHPs. There is a need for more empirical evidence that goes beyond workforce participation numbers and gender [20].

Even though current research has identified a range of factors that influence AHPs’ job choices, it provides only weak evidence on the relative importance of these factors. Other methods are required to collect, analyse and interpret information about job preferences to inform policy development. Stronger research methods are needed to determine what the true “deal breakers” are for leaving or staying in a rural job. For example, most AHPs report that access to professional development and supervision is a retention factor, but it is not known how many will actually leave their position if access is not provided. Similarly, it is not known if other factors such as increased financial reward can offset the reduced access to professional development and supervision. The aim of this study was to understand the relative importance that AHPs working with people with disability in a region in western New South Wales (NSW), Australia, placed on different job characteristics and their decision to stay and practice in a rural area.

## Methods

### Conceptual framework

Discrete choice experiments (DCEs) methodology is a process for determining the relative value that people place on factors (attributes). DCEs are based on the consumer theory of demand [21]; when faced with different alternatives or choices, an individual will choose the alternative that provides the highest utility (or “happiness”). The random utility model [22] is also relevant, in which respondents engage in “utility maximizing” behaviour. In other words, people are assumed to choose the option that has the highest individual benefit or, in economic jargon, “utility”.

This study used a best–worst scaling DCE, also known as multi-profile case best–worst scaling (BWS) experiment or case 3 [23]. BWSDCEs assume that respondents can easily choose items that are extremes (best and worst, most and least, smallest and largest) in a set of three of more items [23]. Compared to traditional DCEs, BWSDCEs provide larger amounts of data and richer information on relative preferences between alternatives [24]. Another advantage of the BWSDCE is that unlike traditional DCEs, in BWSDCEs, the utility of a single level of one attribute acts as a benchmark and not the entire scenario [25]. This allows us to determine the impact of the level of the attribute.

### Identification and selection of attributes and levels

It was important to ensure that attributes (factors) were relevant, grounded in AHPs’ experiences and realistic in terms of generating policy-relevant results [26]. Attributes and attribute levels were selected via extensive qualitative work including in-depth semi-structured interviews and focus groups with 97 purposively sampled service providers working with people with disability in rural New South Wales, Australia. Interviews and focus groups were digitally recorded and transcribed. A modified grounded theory approach using thematic analysis and constant comparison was used to analyse the data [27]. Content analysis of current policy documents was also conducted. Extensive feedback from stakeholders was included via the project management group, which included senior government managers from the study site and the study reference group, which included middle managers and AHPs from government and non-government agencies and carers of people with a disability. Further team discussion and reference to current policy in the sector were incorporated into the wording of the BWSDCE attributes and levels. A detailed description of the qualitative study and BWSDCE attribute development is provided elsewhere [28]. Six attributes were identified: travel arrangements, work flexibility, professional support, professional development, remuneration (as a loading above current salary level) and autonomy of practice (see Table 1).

**Table 1:** DCE attributes, levels and descriptions

Attribute	Levels	Description
Travel arrangements	• One or less nights away per month	Travel that requires overnight stays away from home
	• Two or three nights away per month	
	• Four or more nights away per month	
Flexibility	• Little or no flexibility in work hours	Ability to negotiate your hours of work
	• Some flexibility in work hours	
	• Very flexible work hours	
Professional support	• Rarely	Profession-specific advice and support
	• Sometimes	
	• Readily	
Professional development (PD)	• Minimal	Opportunity to undertake *formal* professional development activities
	• Adequate	
	• Ideal	
Remuneration	• 5 % above your current salary	Rural salary loading above current salary
	• 10 % above your current salary	
	• 15 % above your current salary	
Autonomy of practice	• Limited capacity for independent professional decision-making	Freedom to use professional judgement
	• Some level of independent professional decision-making	
	• High level of independent professional decision-making	

### Experimental design and choice set construction

A combination of six attributes with three levels each would result in 216 potential scenarios (6^3^ = 216). Because this was too many to present to each individual, the number of potential scenarios was reduced by maximizing the D-efficiency (a measure of efficiency) [29]. The *priors* (coefficients) obtained in the pilot phase were used for the model estimate. The design software package NGene 1.1.1 (ChoiceMetrics Pty Ltd, www.choice-metrics.com) was used to generate the design. The final design consisted of 18 sets. Since the number of choice sets increases the cognitive burden, the 18 sets were blocked into 3 questionnaire versions (blocks 1, 2 and 3), each containing 6 choice sets. The blocks were randomly allocated to the respondents. All versions had an extra choice “fixed” set (set 7) at the end with clearly dominant options to test if respondents understood the task. Set 7 responses were not included in the final analysis.

**Fig. 1:**
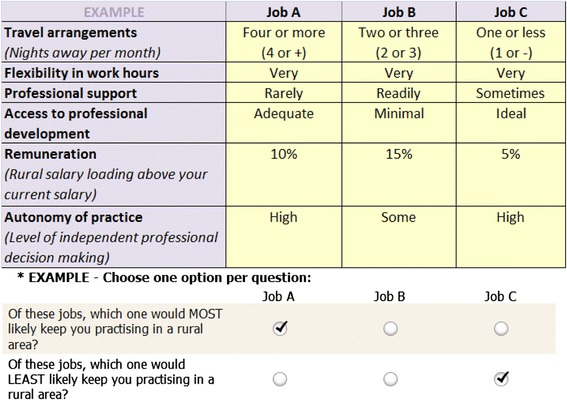
Example of best–worst DCE choice set

Choice profiles were labelled job A, job B or job C. As previously noted, we used a BWSDCE unlike traditional DCEs with a paired decision (job A versus job B and an “opt-out” or “neither” option). In the latter, respondents are asked to choose their preferred option; in this study, for each choice set, respondents were asked to choose which option they preferred: 1) most (“of these jobs which one would MOST likely keep you practicing in a rural area?”) and 2) least (“Of these jobs which one would LEAST likely keep you practising in a rural area”?). This study assumed conditional demand: what employees would value conditional on being in employment. An example of one of the choice sets included in the BWSDCE is provided in Fig. 1.

### Sample and survey administration

Current theory of sampling for BWSDCEs does not directly address the issue of minimum sample size in terms of reliability of the parameter estimates produced in the design. The final sample size required is based upon characteristics of the design itself such as the number of attributes included and the number of choice scenarios presented [30]. The target group for this study was AHPs who were 1) qualified or working as physiotherapist, speech pathologist, occupational therapist or psychologist; 2) working in western NSW; and 3) working with people with a disability. At the time the study was conducted, rurality was defined using the Australian Standard Geographical Classification-Remoteness Area (ASGC-RA) [31]. This ASGC-RA classification is used by the census and Health Workforce Australia. ASGC Remoteness categorizes areas as “major cities”, “inner regional”, “outer regional”, “remote” and “very remote”.

The survey was conducted using an online survey software (SurveyMonkey®). Several recruitment strategies were used to identify potential participants. The invitation email containing a link to the questionnaire was sent to AHPs who had previously participated in the qualitative study conducted as part of the larger study and who had agreed to further contact from the researchers [32]. Government and non-government managers were sent the invitation, which they distributed to AHPs employed at their facilities or services. Email addresses were also gathered from public listings of AHPs via the internet, professional association websites and the yellow pages directory. One of the authors (RC) tracked all the invitations sent. Five email reminders were sent to potential participants during the survey’s 3-month “live” period.

The questionnaire asked about various aspects of the AHPs’ current employment, their workforce preferences and factors that may influence their decision to practice in a rural area. The questionnaire was presented in six sections. Sections 1 and 6 included socio-demographic information, such as gender, age and country of birth, qualification, employment characteristics and income. Section 3 enquired about job satisfaction, work practice options and caseload and also included the BWSDCE. In the BWSDCE, respondents had to complete six choice sets comparing three hypothetical jobs (A, B and C).

### Pilot-testing

To ensure that the language was appropriate and meaningful, the instrument was piloted twice with 15 AHPs employed in roles providing services to people with disability in non-metropolitan areas outside of the area studied. This process also provided an opportunity to determine if respondents understood the attributes and levels. The final instrument was also reviewed by the reference group advising the larger project.

### Data analysis

Choice (0, 1) was the dependent binary outcome, reflecting whether the respondent chose job A, B or C. Choice was coded as “1” whether a best attribute level or a worst attribute level was chosen and as “0” for the remaining (non-chosen) for a particular choice set and individual. The independent variables (attribute levels) were then coded with “1” for a potential best attribute level, “−1” for a potential worst attribute level (to reflect the reciprocal relationship between most and least probabilities [24]) and “0” otherwise. Effects coding was used as it is particularly well suited for BWSDCE since “attribute impacts are estimated separately from the utility level scale values, allowing both comparisons of attribute impact and significance of level scale values to be estimated directly” (P.4) [33].

The model was estimated using conditional logistic regression, assuming the choices of best and worst were made sequentially [24]. In other words, we assumed that respondents always select the *Best* option prior to selecting the *Worst* option. In this study, we had three alternatives (job *A*, *B* and *C*). The probability of observing the preference order *A* > *B* > *C* is modelled as the probability of choosing *A* as best from the set (*A*, *B*, *C*) multiplied by the probability of choosing *B* as worst from the remaining alternatives (*B*, *C*). This is expressed as:

1 $$ P\;\left(\mathrm{ranking}\;A,\;B,\;C,\;D,\;E\right)=\frac{e^{V_A}}{\underset{j=A,\;B,\;C}{{\displaystyle \sum {e}^{V_j}}}}\ *\ \frac{e{-}^{V_C}}{\underset{j=B,\;C}{{\displaystyle \sum {e}^{-{V}_j}}}} $$

Base cases (lower attribute level) were excluded from the conditional regression model. Standard errors were adjusted for clustering of preferences by the respondent. To account for preference heterogeneity between respondents, a latent class model (LCM) was developed. This model can identify the number of classes or segments in a population. The LCM model allows preferences to vary between respondents, assuming that an individual’s decisions depend on factors that are both observed (for example, job attributes and socio-demographic characteristics) and unobserved (for example, attributes and perceptions about a specific job) [34–36]. A backward selection model was used to determine the most appropriate socio-demographic characteristics to include in the model. LCM was performed using Latent Gold version 5.0 (Statistical Innovations Inc., Belmont, MA). Other analyses were conducted using Stata 11.0 for Windows (StataCorp LP, College Station, TX).

## Ethics

This study was approved by the Human Research Ethics Committee of The University of Sydney (Protocol No. 11-2011/14336) and the University of Western Sydney H9446*.*

## Results

**Fig. 2:**
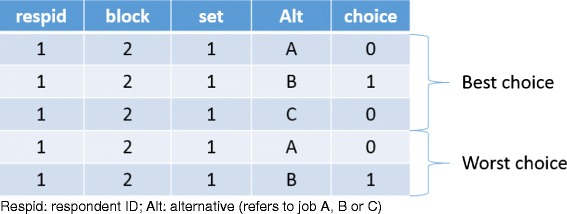
Best–worst data set-up example

Email invitations were sent to 429 AHPs in western NSW. A total of 218 people completed the survey; the response rate was 51 %. Of the 218 people who completed the survey, 7 respondents did not meet the inclusion criteria and 12 (6 %) only partially completed the survey, resulting in a total of 199 useable surveys. Any AHPs working in private practice who stated they would not leave private practice (*n* = 20) were not shown the BWSDCE. Only respondents that completed all choice sets were included in the final sample (*n* = 165). For each choice set, respondents made two choices, 1 = best and 2 = worst. (See detail information in Fig. 2.) Since every respondent completed 6 choice sets (6 × 5), this yielded 4950 best/worst choice observations.

Table 2 summarizes the baseline characteristics of the 165 respondents. Their average age was 38 years (range 23 to 68), 94 % were female, 92 % were born in Australia, 69 % were married or in a *de facto* relationship and 44 % had dependent children. Almost half were occupational therapists, and the mean time since qualification was 10 years (SD 11.3).

**Table 2:** Characteristics of respondents

Characteristic	Frequency	Percent
Mean age, year (SD)	36.0	11.70
Gender		
Female	155	93.9
Allied health profession		
Occupational therapist	77	46.7
Physiotherapist	24	14.5
Speech pathologist	40	24.2
Psychologist	15	9.1
Therapy assistant	9	5.5
Employed by		
Specialists disability government organization	42	25.4
Health	78	47.3
Education	6	3.6
Non-government organization	29	17.6
Private	7	4.2
Other^a^	3	1.8
Country of birth		
Australia	151	91.5
Marital status		
Single	43	26.1
Separated or divorced	7	4.2
Married or *de facto* relationship	113	68.5
Widowed	2	1.2
Dependent children		
No	92	55.8
Mean years in current position (SD)	5.2	6.7
Mean years living in rural area (SD)	7.0	8.5
Family ties in the area		
Yes	107	64.8
Employment status		
Full time	94	57.0
Part time	69	41.8
Other^b^	2	1.2
Personal income^c^		
less than $20 000 per year	6	3.6
$20 000–$39 999 per year	35	21.2
$40 000–$59 999 per year	53	32.1
$60 000–$79 999 per year	38	23.0
$80 000 or more	32	19.4
Did not answer	1	0,6
Annual household income^c^		
less than $20 000 per year	5	3.0
$20 000–$39 999 per year	7	4.3
$40 000–$59 999 per year	32	19.5
$60 000–$79 999 per year	23	14.0
$80 000–$99 999 per year	27	16.5
$100 000–$149 999 per year	40	24.4
$150 000 or more	30	18.3
Did not answer	1	0,6

^a^Other included: disability employment services

^b^Other included: casual, temporary

^c^In Australian dollars

Since this is the first survey conducted with this group of AHPs working with people with disability in rural Australia, we cannot determine if the sample is representative. However, the sample was compared against census data for this same group of AHPs in western NSW and AHPs living in other rural and remote areas of NSW [37, 38]. It is important to highlight that census data in Australia combines speech pathologists with audiologists. Compared to the census data, participants in our sample were more likely to be working part-time, married or in a *de facto* relationship, born in Australia and to have lower personal income. Occupational therapists were over-represented in our sample.

Almost all respondents (96 %) passed the rationality test that was included in the questionnaire (set 7 with a dominant option). Blocks were almost equally completed. Table 3 contains the results of the conditional logistic estimation model. Statistically significant coefficients (*β*) indicate the importance of that attribute for influencing preferences and determining overall utility. Coefficients with positive signs indicate that as the level of the attribute increases so does the utility. BWSDCE allows us to determine the value of the attribute levels. For this group of AHPs, “high autonomy of practice” is the most valued attribute level, followed by “travel arrangements: one or less nights away per month”, “travel arrangements: two or three nights away per month” and “adequate access to professional development”. On the other hand, the least valued attribute levels were “travel arrangements: four or more nights per month”, “limited autonomy of practice” and “minimal access to professional development”. Except for “some flexibility”, all other attributes had a statistically significant influence on AHPs’ job preferences.

**Table 3:** Results from conditional logit regression

Attributes	Coefficient (*ß*)	Std error	*P* value	95 % confidence interval	
Travel arrangements					
(nights away per month)					
Four or more^a^	−1.518	0.317	0.000*	−1.835	−1.201
Two or three	0.714	0.073	0.000*	0.571	0.857
One or less	0.804	0.080	0.000*	0.647	0.961
Flexibility in work hours					
Little^a^	−0.521	0.028	0.000*	−0.549	−0.493
Some	0.028	0.064	0.660	−0.098	0.155
Very	0.493	0.070	0.000*	0.355	0.630
Professional support (available)					
Rarely^a^	−1.005	0.366	0.006*	−1.371	−0.639
Sometimes	0.357	0.082	0.000*	0.197	0.517
Readily	0.648	0.084	0.000*	0.484	0.813
Access to PD					
Minimal^a^	−1.291	0.140	0.000*	−1.431	−1.151
Adequate	0.649	0.078	0.000*	0.495	0.802
Ideal	0.643	0.086	0.000*	0.473	0.812
Remuneration					
(rural loading)					
5 %^a^	−0.709	0.336	0.035*	−1.045	−0.373
10 %	0.279	0.066	0.000*	0.150	0.408
15 %	0.431	0.063	0.000*	0.306	0.555
Autonomy of practice					
Limited^a^	−1.386	0.224	0.000*	−1.610	−1.162
Some	0.555	0.076	0.000*	0.407	0.703
High	0.831	0.080	0.000*	0.673	0.989
Pseudo *R* ^2^	0.069				
Log likelihood	−1650.9158				
Number of respondents	165				
Number of observations	4950				

PD: professional development; *: significant at 5 %

^a^Using effects coding, L-1 levels are calculated using the regression model; the missing level is obtained from the negative of the sum of all other coefficients

**Fig. 3:**
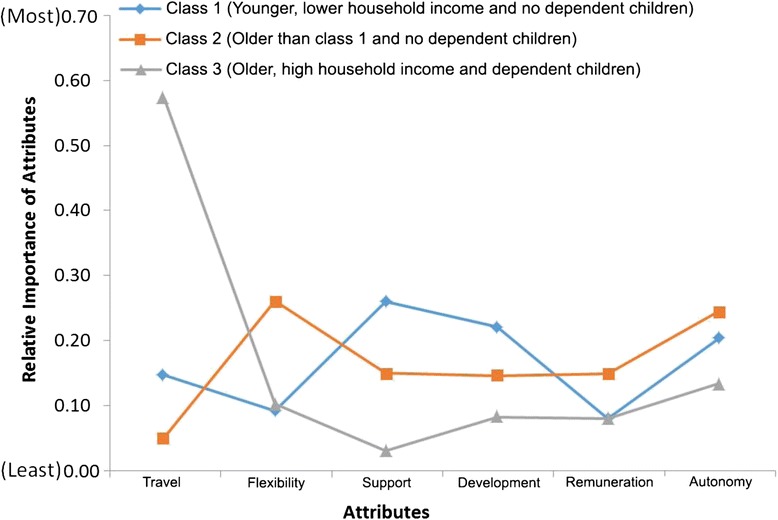
Relative importance of attributes

One respondent did not provide household income and so was excluded from the LCM analysis, leaving a final sample of 164. The log likelihood, the Akaike Information Criteria (AIC) and the Bayesian Information Criteria (BIC) were used as a guide for the number of classes to retain. The following covariates were included in the model: age (as a continuous variable), annual household income and having dependent children (as dichotomous variables). The ideal number of classes was determined when adding another class did not significantly improve the model fit. The class-specific preference estimates are presented in Table 4. The relative importance of the six job attributes to each class is illustrated in Fig. 3. Attributes illustrated in Fig. 3. They are presented as re-scaled values of the maximum effects (that is, values are on a scale of 0 to 1) and in each latent class add up to 1. This allowed us to compare each attribute on the same scale and identify the most important attribute (not the level) between and within the classes. For example, “autonomy of practice” is an important factor for all classes. “travel” is the most important factor for class 3 and “professional support” the least important attribute in this same class. In fact, “travel” is the only attribute where class 2 exceeds the other classes. Class 2 has a stronger relative preference for “flexibility”, and class 1 is sensitive to “professional support” and “professional development” compared to the other three classes.

**Table 4:** Class-specific preference estimates

	Class 1		Class 2		Class 3	
	Coef	S.E.	Coef	S.E.	Coef	S.E
Attribute						
Travel (nights away per month)						
Four or more	−0.5867*	0.108	0.1435*	0.150	−1.9934*	0.182
Two or three	0.2539*	0.070	0.1238*	0.093	0.4484*	0.101
One or less	0.3328*	0.091	−0.2673*	0.149	1.5450*	0.154
Flexibility in work hours						
Little	−0.2909*	0.118	−1.1207*	0.137	−0.4031*	0.131
Some	0.0116*	0.074	0.1120*	0.097	0.1715*	0.111
Very	0.2793*	0.093	1.0087*	0.128	0.2316*	0.119
Professional support						
Rarely	−0.9648*	0.111	−0.5421*	0.124	−0.0875*	0.153
Sometimes	0.3093*	0.080	−0.1366*	0.107	0.1022*	0.125
Readily	0.6555*	0.090	0.6787*	0.111	−0.0147*	0.133
Development (access to CPD)						
Minimal	−0.8530*	0.123	−0.7897*	0.151	−0.2265*	0.136
Adequate	0.3264*	0.071	0.3994*	0.112	−0.0535*	0.118
Ideal	0.5266*	0.098	0.3902*	0.134	0.2799*	0.120
Remuneration						
5 % above	−0.2534*	0.069	−0.6195*	0.121	−0.2248*	0.109
10 % above	0.2430*	0.067	0.0235*	0.093	−0.0408*	0.122
15 % above	0.0104*	0.069	0.5960*	0.120	0.2656*	0.101
Autonomy of practice						
Limited	−0.7652	0.106	−0.9937*	0.137	−0.3517*	0.144
Some	0.2667	0.072	−0.0062*	0.118	−0.1212*	0.126
High level	0.4986	0.086	0.9999*	0.170	0.4729*	0.115
Covariates						
Constant	3.4202*	0.983	3.1409	0.991	2.7213	0.997
Dependent children	−0.4654	0.178	−0.1577	0.162*	0.6231	0.191*
Class probability yes^a^	0.25		0.39		0.75	
High household income	−0.2306	0.150	−0.0508	0.144	0.2814	0.151
Class probability^a^	0.29		0.42		0.62	
Age	0.0063	0.015	0.0069	0.013	−0.0132	0.018
Mean age	36.0		38.0		40.2	
Average class probability	0.4038		0.3078		0.2884	
Log-likelihood	−1433.357					
AIC	2.96					
BIC	3.11					
Pseudo *R*-squared	0.33					

*: significant at 5 %

^a^Proportion ascribed to each class

The class probabilities indicated that class 1 contains 40 %, class 2 contains 31 %, and class 3 contains the remaining 29 % of the subjects. The estimated coefficients for each latent class had the expected sign and in most cases were significant (Table 4). AHPs in the first class highly valued “professional support” and “professional development”. These AHPs were most likely to be younger (mean age 35.6 years), have lower household income and no dependent children.

Class 2 respondents valued “flexibility” and “autonomy of practice” and infrequently chose “travel”. In this class, there is also a change in the significance of coefficients. Respondents in this group were more likely to have no children and had a mean age of 38.0 years. Finally, respondents in the third class placed the highest value on “travel” and “autonomy of practice”. These AHPs were more likely to have dependent children and high household income and were older compared to classes 1 and 2 (mean age 40.2 years). Throughout the results, it can be observed that “remuneration (as a rural salary loading)” is consistently a lesser valued attribute.

As indicated by differences in sign, magnitude and significance of the coefficients, there is significant heterogeneity in preferences across classes. Significant preference heterogeneity was observed in flexibility (Wald test *P* < 0.01).

## Discussion

This is the first study to apply the best–worst multi-profile DCE methodology to explore the job preferences of AHPs working with people with disability in rural Australia. There is limited research focusing on this group of AHPs (occupational therapists, speech pathologist, physiotherapists and psychologists) who work in rural areas and with people with disability [39].

“Autonomy of practice defined as: freedom to use professional judgement” was the most influential of the six job attributes. This was supported in survey comments expressing frustration when they were not able to do what respondents described as “best for their clients”. Importantly, the definition of autonomy as freedom to use professional judgement differs from what has been described in previous studies where autonomy has been defined as the capacity to choose the most suitable job option [40–42].

Nonetheless, previous researchers have also highlighted autonomy as a valued job attribute that motivates occupational therapists and other AHPs (including the professions in this study) to enter private practice and that has a substantial impact on job satisfaction [43, 44]. Independence in clinical decision-making appears critical to these groups of professionals. This finding is not unexpected given that we know people enter allied health careers to “help others”; complete rigorous four year undergraduate or two year masters degrees; and are trained in evidence based practice and research. Removal of autonomy for decision-making, in partnership with people with disability and their carers, restricts AHPs’ opportunities to use the skills they developed through study and experience. It also is in conflict with the best practice in person- and family-centred care. Finally, restricting autonomy is known to reduce job satisfaction.

Contrary to other studies of medical practitioners and nurses [45–47], remuneration was not the main attribute (factor) that will keep these groups of AHPs practising in a rural area. Possibly this is because, in Australia, rural loading and incentives are mainly given to medical practitioners and dentists [48, 49] or to nurses [50] to practise in regional and remote communities. Thus, AHPs do not expect to receive loadings. Furthermore, AHPs working in disability specialist services and non-government organizations have lower wages compared to those in the health- and aged-care sectors [10]. Taken together, these results suggest that factors other than remuneration are more critical in the retention of rural AHPs, including those employed in the disability sector. Future research should investigate the importance of remuneration in the recruitment of rural AHPs since it is well known that there are differences in recruitment and retention factors [3, 44, 51].

Not surprisingly, younger AHPs preferred *professional support* and *continuing professional development* (CPD) (*described as access to CPD*). Lack of professional support and limited career structures have also been described as barriers in previous studies [17, 18, 52, 53]. On the other hand, new AHP graduates often move to rural areas in search of employment opportunities and adventure [3]. Our study, like others, suggests that access to CPD and professional support is particularly crucial in retaining AHPs in an early career stage [54].

Another important factor in the results is a nuanced understanding of the impact of travel on retention. Our qualitative research revealed that it was not distance travelled, *per se*, that was problematic for retention. Rather, it was nights required to be spent away from home [55]. Our findings nuance this further to suggest that nights away from home is an important factor for mid-career AHPs with dependent children and less of a factor for early and late career AHPs. Hence, a major finding from this study is that a “one size fits all” approach to retention policy for AHPs is unlikely to be successful. Policy derived from evidence would result in job descriptions that are tailored to the career stage for AHPs [54].

The results of this study address a gap in our understanding of AHPs’ job preferences. AHPs are important members of the rural health workforce. Changes in skill mix and retention in rural areas will continue to fuel policy debate and development in Australia and elsewhere. Labour supply decisions are influenced by a complex mix of personal preferences for work, leisure, family and lifestyle, the economic and non-economic incentives embedded in the way the system is financed and organized, the culture of practice, and long-term trends in demand, demographics and the composition of the workforce [56]. This study provides empirical evidence on the characteristics that AHPs value most in their professional positions in order to increase retention.

Policymakers and others should be aware that AHPs working with people with disability value autonomy of practice and may be less inclined to respond to remuneration incentives. While limited travel (that is, nights away from home) is important to AHPs with dependent children, younger AHPs are more motivated by professional support and access to CPD. The latter findings are consistent with those of previous researchers.

Compared to other survey studies with other AHPs, our response rate is relatively high [4, 18, 57]. The authors built networks and rapport with participants within the first phase of the study. The high-response rate may also highlight the relevance of the study to participants and their desire to express their opinions on the subject.

## Strengths and limitations

Latent class analysis is an innovative and effective tool for identifying and categorizing heterogeneity of preferences amongst respondents. To our knowledge, this is the first study to quantify AHPs’ preferences for job attributes using multi-profile case best–worst scaling with a latent class analysis. This information can be useful for human resource policymakers. Nonetheless, this study had limitations. Although some characteristics (such as age, gender and income) of our respondents were similar to those of AHPs based in rural western NSW (as per census data), we cannot rule out selection bias.

As is the case for all surveys, terminology and framing of questions is particularly important. Even though all terms in the BWSDCE were defined (see Table 1), respondents could still misinterpret the meaning of questions. For example, “Flexibility” was defined as work flexibility arrangements but could have been interpreted as being flexible personally. We also adopted the sequential best–worst decision rule; we assumed worst and best were opposites and also chosen one after the other (that is, participants chose best and then worst). But this is one of several possibilities, and respondents could have picked both best and worst at the same time (maximum difference (max diff model)). Finally, our BWSDCE design maybe criticized as unrealistic as we did not include an “opt-out” option. The BWSDCE is a conditional demand model and does not provide information on the attractiveness of the hypothetical job relative to the AHP’s current position. Some researchers have argued that having “opt-out” or “neither” options is more realistic [26, 58]. In actuality, health professionals have options, including their current job (*status quo*) or moving to an urban area or withdrawing from the health workforce. Further research could use the best–worst task as part of a wider choice experiment incorporating an “opt-out” option (Would you accept the job chosen as most likely to keep you practicing in a rural area?). Nonetheless, this BWSDCE has provided information at the attribute level, and it is the first time this methodology has been used to explore workforce preferences of AHPs. The other workforce study using BWSDCE explored job preferences of students and new graduates in nursing and also assumed conditional demand [59].

## Conclusions

This study allowed the identification of factors that contribute to AHPs’ employment decisions such as staying and working in a rural area. Use of these findings to tailor position incentives to the characteristics of the desired workforce in rural areas may increase retention of AHPs. Research to inform workforce policy that supports rural disability service delivery is important given the national shortage of allied health therapy services outside of metropolitan centres and the likely growth in demand due to the introduction of the NDIS [14]. The results of this study make a contribution to the existing (mostly descriptive and cross sectional) literature on AHPs in Australia and elsewhere.

## Implications for research and practice

Further research could compare the stated preferences of AHPs with their current situation or assume different decision rules or compare the use of BWSDCEs versus traditional DCEs. For practice, it will be important to explore what motivates these groups of AHPs to work with people with disability.

## Abbreviations

AHPs: Allied health professionals
NDIS: National Disability Insurance Scheme
HWA: Health Workforce Australia
AHWAC: Australian Health Workforce Advisory Committee
NSW: New South Wales
DCE: Discrete choice experiment
BWS: Best–worst scaling
LCM: Latent class model
AIC: Akaike Information Criteria
BIC: Bayesian Information Criteria
CPD: Continuing professional development
